# Supplementary material for: Patterns of Intron Gain and Loss in Fungi
Source: PLoS Biol. 2004 Nov 30;2(12):e422. doi: 10.1371/journal.pbio.0020422 (PMC532390; doi:10.1371/journal.pbio.0020422)
Supplement: Table S1 — Also available at http://genes.mit.edu/NielsenEtAl/. (4.3 MB ZIP). [file pbio.0020422.st001.zip › NielsenEtAl/html/1260.html]

AN4057.1.NCU08995.1.MG05298.1.FG04151.1


```
 CLUSTAL W (1.82) Multiple Sequence Alignments - Introns Inserted


Sequence 1: NCU08995.1	599 aa
Sequence 2: FG04151.1	622 aa
Sequence 3: MG05298.1	662 aa
Sequence 4: AN4057.1	663 aa
Alignment Length: 710 aa
Number Identitical Residues: 175 aa
Alignment Score (without introns) 10624


MG05298.1 	----------------MSGAIEERLAQVLGRTNIAAHARYIIP-ALLLTMAASAPLLIYK
NCU08995.1	------------------------------------------------------------
FG04151.1 	-------------------------------------------------MAAPAEVVDVV
AN4057.1  	MVGCGLLSSLLQRANSARSNKRRRFRRSLSDVHKGSGGTVTSSDEDGYESSESWQHVAET
          	  ...  ::  . :.:  .          .    .: .    ..      : .       

MG05298.1 	RRSTTAGSSQHAGDD1P~ESTDPPLLQKHSEIKSYQTSHFTYPGVRVFYRRHPKADQLPS
NCU08995.1	---------------~-~--MAPQNLKVLTDNTSHRSRGHGSTTS----RQKQRDQNIHH
FG04151.1 	RDAVDS-------TD~P~HLSDPPLLRDHSEFKTYESRGFKYSGIRIFYRQHAKADQLPK
AN4057.1  	RQNQEVHQKGDEPQD~A1VNTDPGLLKKHSLYLSYKTSVAEYPSIRTFNRPHPQMDKLPT
          	       ..     . .     *  *:  :   ::.:     .      * : : :::  

MG05298.1 	TPAPIPLLVFVHGLGGSAAQFTSLLTSLIHVSSCLAIDLPG~CGLSEYAPKDWDAYKTEA
NCU08995.1	V--EVYLSRPAYLLSAPPTGRPSSKVTGADTT-------TR1CGRSEFSVRQWEAYTTDA
FG04151.1 	P--PLPLLVFLHGLGGSVAQFHPLLCSLVDEAPCLAIDYPG~CGRSEFSVTDWAAYTTEA
AN4057.1  	TPSPIPLLVFVHGLGGSLAQFNHLLTSLSNVGPCFGIDLPG~CGLSSFAPTAWDAYTIEA
          	 .: : *    : *... :       :  .  .. . . .  ** *.::   * **. :*

MG05298.1 	LVELLDTIIHDYRDKTTDQDVVLIGHSMGTALAARLASKTVLNPASISRHVVGLVAICPP
NCU08995.1	LVELLETIIEDYREKEDGQGVVLIGHSMGASLAALLASPRLPPRTQLHDHVAGLIAVCPT
FG04151.1 	LVELLEIIINDYRDKGAGQRVVLIGHSMGTALSARLANTGLRHTTDLSHHVVGLIAICPV
AN4057.1  	LAELLATAIDRHRDKEAGQKVVLIAHSLGCSLSAMLTSSTSPLKHELKDHILGLVAICPR
          	*.***   *. :*:*  .* ****.**:* :*:* *:.       .:  *: **:*:** 

MG05298.1 	SEPPPVKQAGLARKFLSIPTPILDLYRAWDKRGGLESASVSRFIGADASKEAKKLQYRFN
NCU08995.1	SGFSSQKQIMVLTALLWVPEIIFNLWRAWDKRGGSQSASVRRFVGADADPEAKRLQDLYN
FG04151.1 	ANPPNESLTRWAKIAMWIPGWLFDLWRAWDGRGGPESASVKRFVGQGADKATRKMQYRFN
AN4057.1  	ASPPSPKEVSSHRRLLYIPDSIFNLWRRWDRRGGLYSNSVNRLVGAGADEETRSLQIRFN
          	:  .  .        : :*  :::*:* ** ***  * ** *::* .*.  :: :*  :*

MG05298.1 	CQSRTPVWRRMAYGFLPTYH--DGDTHGGFAGREVWKGLKIPVYLVAGRDDHITKPSDAE
NCU08995.1	NQAITSVWRRMAWGCLPTHV--KGVTKGGLPSKETWEKLNVPVFLVGGEDDRVTPPQEIA
FG04151.1 	KQSRTPVWRRMANGALPVYN--DGKPTGGLPTLDTWAGLDIPVFLIAGERDNVVSPKEAE
AN4057.1  	KQSKTPVWKRMVWGTLPSYSGPNSKPISGLPGQEVWAGVKTPILLIGGESDMVTRPVELQ
          	 *: *.**:**. * ** : .... . .*:.  :.*  :. *: *:.*. * :. * :  

MG05298.1 	RIREWLSEDVDSTASPRDG---PIIVDSAAPLEVPIERPDTRGKSRIEDLT---------
NCU08995.1	KIRDFLQEVKTTPDTISPG--TSVLADSAAPVDINAA------ASKIDAMTSLGQGEENT
FG04151.1 	KIVKVLDPDKTSPESEDGQEMHEIIVDTVARVNISTR------PQSISDLT---------
AN4057.1  	KLLRALGDTGNDK-----------TMDEDADGSVAAS-----------------------
          	::   *                    *  *  .:                          

MG05298.1 	--DEDFQRARHRVSLDDLQE----DPSTPREGSTEIPTSPLRPRRSVELIILPSPATHAL
NCU08995.1	RESEDQKPVHRTQVAEPEEG--ADDPVTPTEQAASLPSLSLHRKKIVRSIIMGKPATHAL
FG04151.1 	--DADFMKVKSPHVETPPEGGSSTDPSTPNETLTELPPQPSHPKKIVQSFVMPAPATHAV
AN4057.1  	----EASMLPDSLAHEEKLG---IEPQLKEKVTNESNGLP-RSKRSVKTVILPAPASHAL
          	    :                   :*    :   .    . : :: *. .::  **:**:

MG05298.1 	LYAPSTARMLAGLVSDFLSTSISGRLSLGWQLQYLSKEGKWDVKNLAKWKNVEPVSDPIA
NCU08995.1	LYTPSTVRILAGLISDFMVVHITGRLDLGWQLQYLSRDGKWDVKNLQKWQAVEPVSEPIG
FG04151.1 	LYTPCSVRAVAGLISDFLATNVTGRLSLAWQLQYLSREGKWDVKNLNKWKSVNPVSDPIG
AN4057.1  	LYDRATYRTLAGIIQDFVSQHADHRLNLGWQLQYLNTSGKWDVKNLAKWKKVPPVSDRIA
          	**  .: * :**::.**:      **.*.******. .******** **: * ***: *.

MG05298.1 	G----IFRAMKTLREVDEIHSPKSFVENWGHSIRDIIDISHDTPVYDPAGLEAGGIRYHK
NCU08995.1	G----IFRAIKTLREVDEDHSPSEFVKKWGGVIKDVIDISHDNPVYNPQGLEKGGIHYHK
FG04151.1 	PPGKPIFRALKTLREADDVHCPEEFVSKWSSIVKDVIDISKDQPVYDPRGLERAGIRYHK
AN4057.1  	N----TFVALKMLREVDEEHNPVLFSKAHRDNIYTVIDISHESPVYNPASLEAGGIHYQK
          	      * *:* ***.*: * *  * .     :  :****:: ***:* .** .**:*:*

MG05298.1 	FPSVSKIPPTPEEVEAFIALVDKVRAEQREKLQG-------DMKAAVAVHCHYGF0NRTG
NCU08995.1	FPTVSKVPPTDAEIKGFIELVDKVRDEQKERAKRENWG----EEHYIGVHCHYGF~NRTG
FG04151.1 	FPTVSKIPPLSHEIQDFIKLVDDIRQSQADRALTEEWS--DPEQCVIGVHCHYGF~NRTG
AN4057.1  	YPTVSKIPPTPDEVRDFIALVDRLQKEITEKMEKSNTSGAARLRPVVGVHCHYGF~NRTG
          	:*:***:**   *:. ** *** :: .  ::   .. ..:   .  :.******* ****

MG05298.1 	FFIVCYLIERVGFSVQAAIDEFARARPKGIRHQHFLDQLFVRYSSLDMKRKQEQ
NCU08995.1	FFLVCYLVERCGYTPEAAIEHFAQSRPKGIKHAHFKDRLYVRYSGLRSEAIAEH
FG04151.1 	YFIVCYLVEKCGFDVQEAIETFAKARPNGIRHSHFLDRLYVRYNV---------
AN4057.1  	FLIVSYLIERCGFGVQEAIDEFEKRRPPGIRHAHFIDTLFVRYCVGLKRAPTL-
          	:::*.**:*: *:  : **: * : ** **:* ** * *:***
```
